# Japanese translation of lindop Parkinson’s disease mobility assessment (LPA): validity and reliability study

**DOI:** 10.1007/s10072-026-09370-0

**Published:** 2026-09-08

**Authors:** Seira Taniguchi, Kohei Marumoto, Masaru Narita, Yuki Kawami, Tsuyoshi Kozuki, Takeru Matsuda, Kazuma Miura, Lindun Ge, Yohei Okada, Kensuke Ikenaka

**Affiliations:** 1 Department of Neurology, The University of Osaka Graduate School of Medicine, Osaka, Japan; 2 Kinesiology and Health Sciences, University of Waterloo, Waterloo, ON Canada; 3 Hyogo Prefectural Rehabilitation Hospital at Nishi-Harima, Hyogo, Japan; 4 Neurological Rehabilitation Center, Hokkaido Neurological Hospital, Sapporo, Japan; 5 Task Force on Parkinson’s Disease, Japanese Society of Neurological Physical Therapy, Tokyo, Japan; 6 Neurorehabilitation Research Center, Kio University, Koryo-cho, Japan

**Keywords:** Parkinson’s disease, Functional mobility, Gait, Bed mobility, Validity, Reliability

## Abstract

**Introduction:**

The Lindop Parkinson’s Disease Mobility Assessment (LPA) is a Parkinson’s disease (PD)-specific clinical scale. The LPA was designed to assess core functional mobility in rehabilitation, including gait and bed mobility. This study aimed to develop a Japanese version of the LPA and evaluate its validity and reliability in PD.

**Methods:**

Sixty-two people with PD participated in the validation analyses. The Japanese version of the LPA was developed through a standardized translation and cross-cultural adaptation process. Construct validity was examined using Spearman’s rank correlations between LPA scores and relevant clinical measures. Internal consistency was assessed using Cronbach’s alpha. Inter-rater reliability for video-based assessments was evaluated using intraclass correlation coefficients (ICCs) and quadratic weighted kappa coefficients.

**Results:**

The total LPA score showed very strong correlations with the axial subscore, MDS-UPDRS Part 3, bradykinesia subscore, and Berg Balance Scale (Spearman’s rho = − 0.83, − 0.74, − 0.70, and 0.75, respectively; all FDR-adjusted *p* < 0.0001). Internal consistency was excellent for the total score, gait mobility section, and bed mobility section (Cronbach’s α = 0.95, 0.94, and 0.94, respectively). Inter-rater reliability was excellent for the total score (ICC [2, 1] = 0.97). Item-level agreement assessed using quadratic weighted kappa coefficients ranged from 0.46 to 1.00 (all *p* ≤ 0.009).

**Conclusion:**

The Japanese version of the LPA demonstrated good construct validity and excellent internal consistency and inter-rater reliability for the total score in assessing gait and bed mobility in PD. Further studies are needed to examine its responsiveness and applicability among individuals with more advanced functional impairment.

**Supplementary Information:**

The online version contains supplementary material available at https://doi.org/10.1007/s10072-026-09370-0.

## Introduction

Difficulties of bed mobility, gait and balance are common and debilitating in people with Parkinson’s disease (PD). Bed mobility is a prerequisite for subsequent functional mobility, including sitting up, standing, walking, and other daily activities [1]. Consequently, impaired bed mobility can restrict overall functional independence and increase the need for assistance [2]. In people with PD, impaired bed mobility is known to emerge early in the disease course and become more pronounced with disease progression [3], contributing to reduced independence and increased caregiver burden [4]. While various rating scales with adequate clinimetric properties have been used to assess gait disorders in PD, assessing bed mobility in PD remains particularly challenging due to its complexity including axial and limb movements with multiple strategies possible [5]. A previous review identified three types of objective methods for assessing impaired bed mobility in PD: sensor-based assessments, rating scales, and timed tests. Among these, rating scales were considered the most clinically feasible approach because they have relatively established clinimetric properties and are easy to administer in physiotherapy practice [6].

The Lindop Parkinson’s Disease Mobility Assessment (LPA) is a 10-item PD-specific clinical scale, designed to assess core functional mobility [7]. It consists of two sections: a six-item gait mobility section, and a four-item bed mobility section. The gait mobility section includes the following items: sit-to-stand, unsupported standing, timed up-and-go, turning, and walking-through-doorway, while the bed mobility section includes sit-to-lie, turning to the left and right in bed, and lie-to-sit items. Each item is scored on a 4-point ordinal scale ranging from 0 (worst) to 3 (best). Importantly, the LPA considers not only the quality of movement, but also the time required to complete each task; for instance, the bed mobility item 1 (sit-to-lie) distinguishes between “unaided with ease within 5s” (3 points), “unaided with effort 6 s or more” (2 points), “help of one” (1 point), and “help of two/unable” (0 points). The LPA has been globally used [8–11] and studies have examined its validity and reliability. It also has the practical advantage of being brief and easy to administer in clinical settings [7, 8, 11], as recommended by the European Physiotherapy Guideline for PD [12]. A Brazilian Portuguese version has also undergone cross-cultural adaptation and evaluation of its measurement properties [13]. However, despite its clinical utility, a Japanese version of the LPA is not currently available. Therefore, the aim of the present study was to develop a Japanese version of the LPA and evaluate its validity and reliability in people with PD.

## Methods

### Inclusion and exclusion criteria

People with PD were recruited for this study from two different medical centers in Japan: Hyogo Prefectural Rehabilitation Hospital at Nishi-Harima (i.e., Hyogo center) and Hokkaido Neurological Hospital (i.e., Hokkaido center). All participants were consecutively enrolled from these centers from June 2025 to March 2026, and provided written informed consent. The inclusion criterion was a diagnosis of idiopathic PD based on the Movement Disorder Society (MDS) clinical diagnostic criteria [14]; the exclusion criteria were: (1) the presence of conditions other than PD that could affect gait/balance and bed mobility. This was assessed during study enrollment through an interview with the participant and, where appropriate, family members, together with a review of available clinical information; (2) cognitive impairment that, as determined by a trained physiotherapist during the pre-assessment explanation, prevented participants from understanding or carrying out the required tasks, in accordance with the protocol of the original LPA validation study [7]. No eligibility restrictions were imposed on age or H&Y stage. Following the approach adopted in previous multicenter validation studies of assessment scales in PD, including the MDS-sponsored revision of the Unified Parkinson’s Disease Rating Scale (MDS-UPDRS) and the Non-Motor Symptoms Scale [15–17], the present study was designed to assemble a larger sample encompassing a broad range of motor severity, rather than to compare the two centers as clinically matched cohorts. For descriptive transparency, center-specific demographic and clinical characteristics are presented in the Supplementary Material. In addition, to examine whether recruitment center influenced the construct-validity findings, sensitivity analyses adjusting for recruitment center were performed, as described in the Statistical Analysis section.

### Development and cross-cultural adaptation of the Japanese version of the LPA

We developed the Japanese version of the LPA according to a standardized protocol with prior consent from the original authors, following a cross-cultural adaptation protocol [18]. The process included forward translation, back-translation, comparison with the original English version, and consultation with the original developer. Specifically, the LPA was translated from English into Japanese by a native Japanese-speaking physiotherapist with experience in PD research and rehabilitation (ST). The preliminary Japanese version was back-translated into English by a professional translator at Cactus Communications Co., Ltd., who was a native English speaker, proficient in Japanese, and unfamiliar with the original scale. The back-translation was compared with the original English version, and any discrepancies were discussed and resolved in consultation with the original developer. In the original LPA, the maximum score of 3 points for item 3, the timed up-and-go test, was assigned to completion times of 10–20 s, whereas completion times below 10 s were not explicitly covered by any scoring category. During the translation and adaptation process, with permission from the original developer, the maximum-score category was therefore clarified to include completion times of 0–20 s in the Japanese version. In the present validation sample, 17 of 62 participants completed the task in less than 10 s and fell within this previously undefined range. This clarification did not alter the boundaries between the other scoring categories or reclassify participants whose completion times fell within the categories specified in the original LPA. Details of the Japanese version of the LPA are provided in the Online Resource 1.

### LPA and clinical assessment

All assessments were performed across centers during the “ON” medication state after levodopa intake. The gait and bed mobility sections of the LPA were assessed as part of routine physiotherapy assessment by registered physiotherapists with clinical experience in PD. For the inter-rater reliability analysis, video-based assessments obtained from a subsample of participants at Hokkaido center were independently scored by two physiotherapists, who had 25 and 4 years of clinical experience, respectively. Video recording had been approved at Hokkaido center, and participants provided consent for recording. No recordings were obtained at Hyogo center in accordance with site-specific ethical and personal information protection requirements. General neurological assessments included the Hoehn and Yahr (HY) scale, the MDS-UPDRS Parts 2 and 3. Additionally, the MDS-UPDRS-based axial, bradykinesia, and rigidity subscores were calculated as follows: axial subscore, the sum of items 3.1 and 3.9–3.13; bradykinesia subscore, the sum of items 3.2, 3.4–3.8, and 3.14; and rigidity subscore, the sum of sub-items of item 3.3. Balance performance was evaluated using the Berg Balance Scale (BBS).

#### Statistical analysis

##### Validity

Construct validity was examined using Spearman’s rank correlations between the LPA scores and existing functional measures. Given that the LPA is based on clinical observation and timed performance tasks, clinician-rated motor measures, such as the BBS and relevant MDS-UPDRS Part 3 items, were prioritized as validation measures. For the total LPA score, correlations were examined with MDS-UPDRS Part 3 total score, axial subscore, bradykinesia subscore, and BBS. For the section-level analyses, correlations were examined between the gait mobility section score and the axial subscore, MDS-UPDRS item 3.10 (gait), item 3.11 (freezing of gait), and BBS. Correlations were also examined between the bed mobility section score and the axial, bradykinesia, and rigidity subscores.

At the item level, exploratory correlations were evaluated between each LPA item and clinically corresponding validation measures: gait-related LPA items were correlated with MDS-UPDRS item 3.9 (arising from chair), item 3.10 (gait), item 3.11 (freezing of gait), and item 3.12 (postural stability), and the timed up-and-go item was correlated with the BBS. Bed mobility-related LPA items were correlated with MDS-UPDRS item 2.9 (turning in bed), item 2.11 (getting out of bed, a car, or a deep chair), and the axial subscore. For the turning-in-bed analysis, the scores for the left and right turning items were summed to create a combined turning-in-bed score ranging from 0 to 6.

We primarily focused on the total LPA score and section-level scores, while item-level correlations were additionally examined as supplementary outcomes. To reduce potential circularity, validation analyses were conducted using related but more independent measures of balance and gait function, such as the BBS and relevant MDS-UPDRS Part 3 items, rather than the timed up-and-go time or bed mobility completion time itself.

Spearman’s rank correlation coefficients (rho) were interpreted according to previously reported criteria, with absolute values of 0.30–0.39 considered moderate, 0.40–0.69 considered strong, and ≥ 0.70 considered very strong [19]. Moreover, as a sensitivity analysis, partial Spearman correlations were calculated adjusting for recruitment center to examine whether the construct validity findings were influenced by center membership. For multiple validity correlation analyses, p-values were adjusted using the false discovery rate (FDR) method.

##### Reliability

Internal consistency was examined using Cronbach’s alpha (α) based on the 10 LPA item scores from all participants. For inter-rater reliability, the total LPA score was assessed using the intraclass correlation coefficient (ICC [2, 1]) and Bland–Altman analysis, whereas item-level agreement was assessed using quadratic weighted kappa coefficients to account for their ordinal nature.

##### Floor and ceiling effects

Floor and ceiling effects were examined for the total LPA score and section-level scores. Floor or ceiling effects were defined as occurring when more than 15% of participants achieved the minimum or maximum score, respectively [20].

All statistical analyses and data visualization were performed using R software (version 4.3.1). A p-value of < 0.05 was considered statistically significant.

## Results

### Participants’ demographic information

Sixty-two people with PD from two medical centers were included in the validation analyses and the assessment of internal consistency: 30 from Hyogo center and 32 from Hokkaido center. The mean total LPA score was 22.8 ± 8.6, with mean gait and bed mobility section scores of 13.5 ± 6.0 and 9.2 ± 3.1, respectively. Participants were aged 55–89 years and had H&Y stages 2–5, and MDS-UPDRS Part III scores ranged from 3 to 98. Further demographic and clinical characteristics are presented in Table 1. Of these, 24 participants from Hokkaido center were included in the video-based assessments for inter-rater reliability and item-level agreement, due to site-specific approval and participant consent for video recording. The reliability subsample differed from the remaining 38 participants in levodopa equivalent daily dose and MDS-UPDRS Part II total score; the full comparison is presented in Online Resource 2 (eTable 1).

**Table 1:** Demographic characteristics of the participants (*n* = 62; 34 women, 28 men)

	Mean	Standard deviation (min, max)
Age (years)	75.1	7.2 (55, 89)
Disease duration (years)	8.6	5.1 (0, 25)
Hoehn and Yahr stage (2/3/4/5)	*n* = 14/30/14/4	
LPA total score (range 0–30)	22.8	8.6 (0, 30)
Gait mobility section score (range 0–18)	13.5	6.0 (0, 18)
Bed mobility section score (range 0–12)	9.2	3.1 (0, 12)
Levodopa equivalent daily dose (mg/day)	666.4	321.6 (100, 1450)
MDS-UPDRS Part 2 total (range 0–52)	16.8	12.1 (0, 52)
Item 2.9, Turning in bed (range 0–4)	1.2	1.2 (0, 4)
Item 2.11, Getting out of bed/chair (range 0–4)	1.5	1.2 (0, 4)
MDS-UPDRS Part 3 total (range 0–132)	35.6	17.5 (3, 98)
Item 3.9, Arising from chair (range 0–4)	1.0	1.3 (0, 4)
Item 3.10, Gait (range 0–4)	1.6	1.3 (0, 4)
Item 3.11, Freezing of gait (range 0–4)	0.8	1.2 (0, 4)
Item 3.12, Postural Stability (range 0–4)	2.1	1.4 (0, 4)
Axial subscore (range 0–24)	8.2	5.7 (0, 24)
Bradykinesia subscore (range 0–48)	15.6	9.3 (0, 47)
Rigidity subscore (range 0–20)	8.4	3.3 (0, 15)
Berg Balance Scale (range 0–56)	42.1	17.4 (0, 56)
Mini Mental State Examination (range 0–30)	26.1	3.8 (11, 30)

Values are presented as mean ± standard deviation (minimum, maximum), except for Hoehn and Yahr stage, which is presented as counts. Abbreviations: MDS-UPDRS = Movement Disorder Society-sponsored revision of the Unified Parkinson’s Disease Rating Scale

### Construct validity

**Fig. 1:**
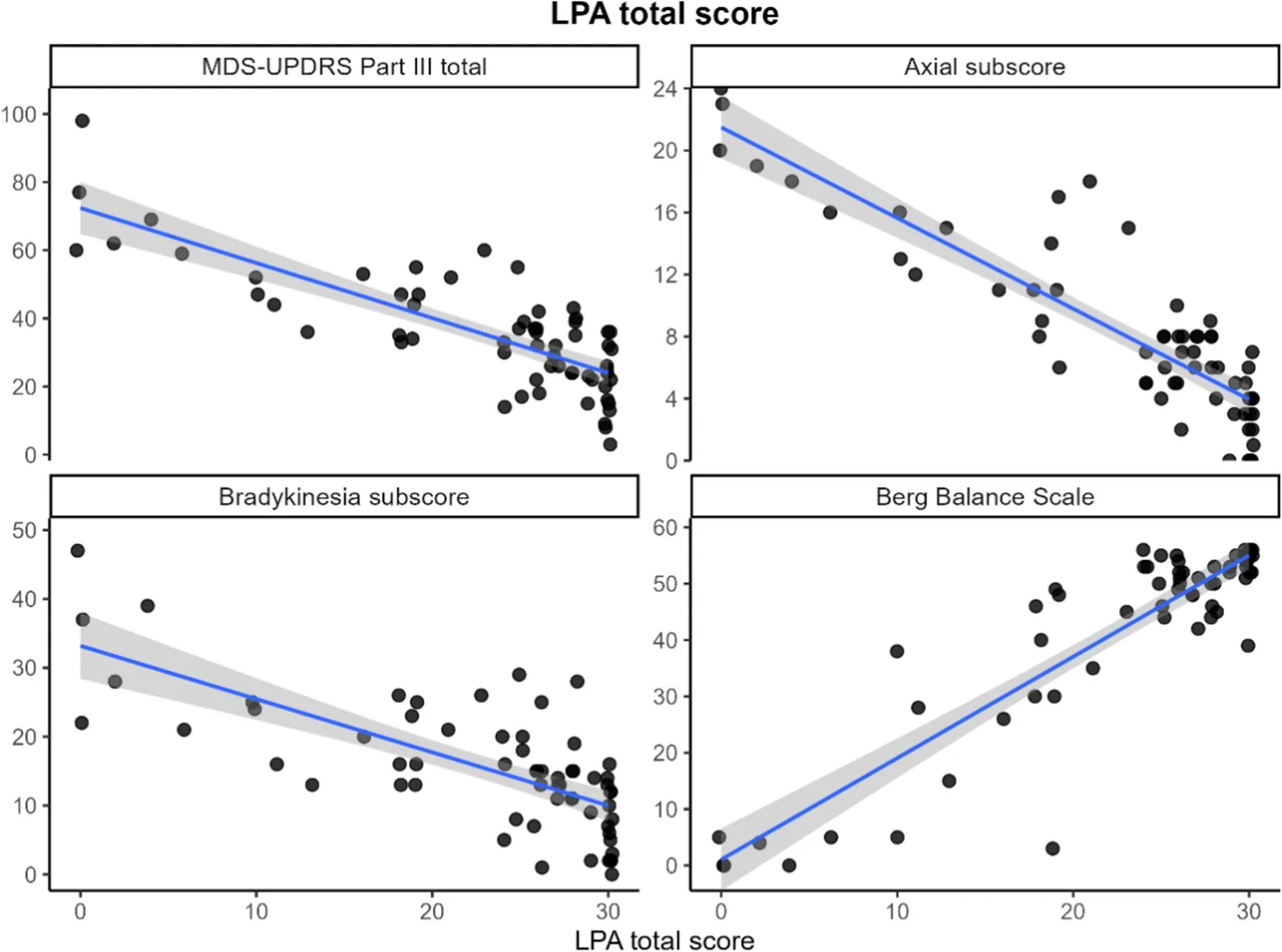
Correlations between LPA total score and relevant clinical measures. The x-axis shows the LPA total score, and the y-axis represents each relevant clinical measure. Each point represents an individual participant’s raw data, and points were slightly jittered horizontally to improve the visibility of overlapping observations. The blue line represents a fitted linear trend line for visualization, and gray shaded areas indicate 95% confidence intervals around the fitted line. Spearman’s rho and p-values are summarized in Table 2

The results of Spearman’s rank correlation analyses are summarized in Table 2, and corresponding total-score scatter plots are shown in Fig. 1.

**Table 2:** Construct validity of the Japanese LPA: Spearman’s rank correlations with relevant clinical measures (N = 62)

LPA scores	Validation measures	Spearman’s rho	FDR-adjusted *p*
**LPA total score**	MDS-UPDRS Part 3 total score	−0.74	< 0.0001
	Axial subscore	−0.83	< 0.0001
	Bradykinesia subscore	−0.70	< 0.0001
	BBS	0.75	< 0.0001
**Gait mobility section total**	Axial subscore	−0.79	< 0.0001
	MDS-UPDRS 3.10 Gait	−0.70	< 0.0001
	MDS-UPDRS 3.11 FOG	−0.52	< 0.0001
	BBS	0.70	< 0.0001
Item 1: Sit-to-stand	MDS-UPDRS 3.9 Arising from chair	−0.80	< 0.0001
Item 2: Unsupported standing	MDS-UPDRS 3.12 Postural stability	−0.57	< 0.0001
Item 3: Timed up-and-go	BBS	0.75	< 0.0001
Item 4: 180° turn, right	MDS-UPDRS 3.10 Gait	−0.60	< 0.0001
	MDS-UPDRS 3.11 FOG	−0.50	< 0.0001
Item 5: 180° turn, left	MDS-UPDRS 3.10 Gait	−0.60	< 0.0001
	MDS-UPDRS 3.11 FOG	−0.47	< 0.001
Item 6: Walking-through- doorway	MDS-UPDRS 3.11 FOG	−0.46	< 0.001
**Bed mobility section total**	Axial subscore	−0.71	< 0.0001
	Bradykinesia subscore	−0.63	< 0.0001
	Rigidity subscore	−0.35	< 0.01
Item 1: Sit-to-lie	MDS-UPDRS 2.11, Getting out of bed/chair	−0.49	< 0.0001
Items 2 and 3: Turn on bed	Axial subscore	−0.63	< 0.0001
Items 2 and 3: Turn on bed	MDS-UPDRS 2.9, Turning in bed	−0.60	< 0.0001
Item 4: Lie-to-sit on bed	MDS-UPDRS 2.11, Getting out of bed/chair	−0.47	< 0.001

MDS-UPDRS = Movement Disorder Society-sponsored revision of the Unified Parkinson’s Disease Rating Scale, BBS = Berg Balance Scale, FOG = freezing of gait, Getting out of bed/chair = Getting out of bed, a car or a deep chair, FDR = the false discovery rate

The total LPA score showed very strong correlations with the axial subscore, MDS-UPDRS Part 3 total score, bradykinesia subscore, and BBS, with the strongest correlation observed for the axial subscore (rho = − 0.83, FDR-*p* < 0.0001).

At the section level, the gait mobility section showed the strongest correlation with the axial subscore (rho = − 0.79, FDR-*p* < 0.0001), followed by MDS-UPDRS item 3.10 (gait; rho = − 0.70, FDR-*p* < 0.0001) and BBS (rho = 0.70, FDR-*p* < 0.0001), while the bed mobility section also showed the strongest correlation with the axial subscore (rho = − 0.71, FDR-*p* < 0.0001), followed by the bradykinesia subscore (rho = − 0.63, FDR-*p* < 0.0001). These section-level correlations are illustrated in the Online Resource 2 (eFig. 1).

At the item level, the sit-to-stand item showed a very strong correlation with MDS-UPDRS item 3.9 (arising from chair; rho = − 0.80, FDR-*p* < 0.0001), the unsupported standing item showed a strong correlation with MDS-UPDRS item 3.12 (postural stability; rho = − 0.57, FDR-*p* < 0.0001), and the timed up-and-go item showed a very strong correlation with the BBS (rho = 0.75, FDR-*p* < 0.0001). The right and left 180-degree turning items showed strong correlations with MDS-UPDRS item 3.10 (gait; rho = − 0.60, FDR-*p* < 0.0001 for both) and item 3.11 (freezing of gait; rho = − 0.50, FDR-*p* < 0.0001 and rho = − 0.47, FDR-*p* < 0.001, respectively), while the walking-through-doorway item showed a strong correlation with MDS-UPDRS item 3.11 (rho = − 0.46, FDR-*p* < 0.001). Further, self-reported bed mobility items in MDS-UPDRS Part 2 showed strong correlations with the corresponding LPA bed mobility items. Specifically, the sit-to-lie and lie-to-sit items were associated with MDS-UPDRS item 2.11 (getting out of bed/chair; rho = − 0.49, FDR-*p* < 0.0001 and rho = − 0.47, FDR-*p* < 0.001, respectively), and the combined turning-in-bed items were associated with the axial subscore (rho = − 0.63, FDR-*p* < 0.0001) and MDS-UPDRS item 2.9 (turning in bed; rho = − 0.60, FDR-*p* < 0.0001). For descriptive transparency, center-specific demographic and clinical characteristics are presented in eTable 2. In sensitivity analyses adjusting for recruitment center, all 23 center-adjusted correlations retained the same direction as the corresponding unadjusted correlations and remained statistically significant after FDR correction (eTable 3). Further details are provided in the Supplementary Material.

The observed correlations should be interpreted with caution in light of the ceiling effects reported below.

## Reliability

**Fig. 2:**
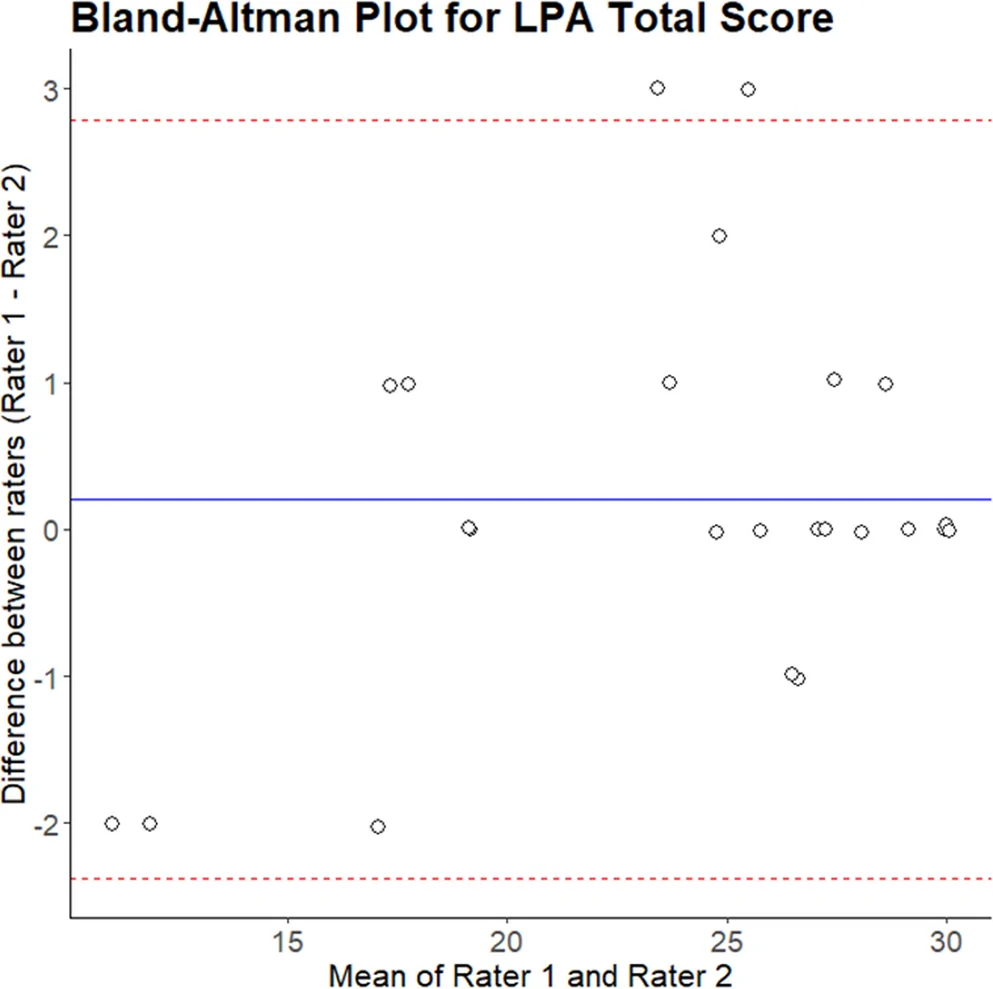
Bland–Altman plot for the LPA total score in the subsample (*n* = 24). The solid line indicates the mean difference between raters (0.21 points), and the dashed lines indicate the 95% limits of agreement (− 2.38 to 2.79 points). Note: Points were slightly jittered horizontally to improve the visibility of overlapping observations

Internal consistency was examined in all 62 participants. Cronbach’s alpha was 0.950 for the total LPA score, indicating excellent internal consistency. Section-specific alpha values were also high for the gait mobility section (α = 0.935) and bed mobility section (α = 0.936). Inter-rater reliability and item-level agreement are summarized in Table 3. The ICC (2,1) for the total LPA score was 0.973, indicating excellent inter-rater reliability. Item-level agreement was assessed using quadratic weighted kappa coefficients, which ranged from 0.464 to 1.000 and were statistically significant for all items (all *p* ≤ 0.009). Bland–Altman analysis (Fig. 2) showed a mean difference of 0.21 points, with 95% limits of agreement ranging from − 2.38 to 2.79 points.

**Table 3:** Inter-rater reliability and agreement for the Japanese version of LPA in the subsample (n = 24)

Measures	Statistic	Value	p-value
LPA total score	ICC(2,1)	0.97	–
*Gait mobility*			
1. Sit-to-stand	QWK	0.54	0.003
2. Timed unsupported stand	QWK	1.00	< 0.0001
3. Timed up-and-go	QWK	0.87	< 0.0001
4. 180-degree turn, right	QWK	0.95	< 0.0001
5. 180-degree turn, left	QWK	1.00	< 0.0001
6. Walking-through-doorway	QWK	0.46	0.009
*Bed mobility*			
1. Sit-to-lie	QWK	0.75	0.0001
2. Turn to left on bed	QWK	0.79	0.0001
3. Turn to right on bed	QWK	0.86	< 0.0001
4. Lie-to-sit on bed	QWK	0.71	0.0003

ICC = intraclass correlation coefficient, QWK = quadratic weighted kappa

### Floor and ceiling effects

Floor effects were not observed for the total LPA score or section-level scores. However, ceiling effects were observed for the LPA total (24.2%), gait mobility section (43.5%), and bed mobility section (29.0%).

## Discussion

### Validity

Overall, the Japanese version of the LPA demonstrated good construct validity at the total, section, and item levels. The total score and gait mobility section score showed very strong inverse associations with axial motor symptoms, while the bed mobility section score showed a very strong inverse association with axial motor symptoms (rho = − 0.71), indicating that lower independence in gait and bed mobility was associated with greater axial motor impairment. The total LPA score was also associated with MDS-UPDRS Part 3, which is consistent with previous LPA validation studies. In addition, the mean total LPA score and age in the present study were comparable to those reported in the original validation study by Pearson et al. (LPA total score: 22.8 versus 22.0; age: 75.1 versus 75.8 years) [7]. Previous validation studies by Pearson et al. and Santos et al. mainly focused on correlations between the total LPA score and global motor severity in PD [7, 13]. The present study extends these findings by examining section- and item-level associations in more detail, showing that LPA scores across gait and bed mobility domains were associated with clinically relevant measures, with the strongest associations observed for axial motor symptoms.

Standardized differences indicated between-center differences in several clinical characteristics, particularly measures of motor severity and balance performance. However, the similarity between the unadjusted and center-adjusted correlations suggests that the principal construct-validity findings were not materially influenced by recruitment center. These correlations should also be interpreted in light of the ceiling effects described below, particularly for the gait mobility section.

In the gait mobility section, the strongest associations were observed with axial symptoms, followed by gait-related clinical measures. This finding is not surprising because four out of six items directly involve gait or turning, while only the walking-through-doorway item directly assesses FOG, which is one of the axial motor symptoms in PD. At the item level, the relatively weaker association with FOG severity may be partly explained by the fact that only 27 of 62 participants had clinically observed FOG based on MDS-UPDRS item 3.11.

In the bed mobility section, the strongest association was also observed with axial symptoms, followed by bradykinesia and rigidity subscores. This finding is clinically plausible as impaired bed mobility in PD is often characterized by difficulty turning in bed [21], which requires axial muscle flexibility and trunk control. In addition, upper limb rigidity may also contribute to impaired bed mobility. As axial dysfunction becomes more pronounced, PD patients may increasingly rely on compensatory upper limb movements, such as pushing with the arms or using the legs to assist turning and rising [22]. However, the present validation study was not designed to determine the relative contributions of limb rigidity and axial dysfunction to reduced bed mobility independence [23]. Further analyses could clarify these relationships, but this was beyond the scope of the present validation study.

### Reliability

The Japanese version of the LPA showed excellent inter-rater reliability for the total score (ICC [2, 1] = 0.973). In addition, the small mean difference in the Bland–Altman analysis suggested no substantial systematic bias between raters. These findings are consistent with the original LPA study, in which Pearson et al. reported good inter-rater agreement, no systematic bias between raters, and 95% limits of agreement within approximately two scale points [7]. Similarly, Verheyden et al. reported high inter-rater reliability for functional mobility items, with ICC values ranging from 0.95 to 0.99 for sit-to-stand, timed up-and-go, and bed mobility [8].

Although most items showed high agreement, the walking-through-doorway item showed the lowest weighted kappa coefficient (0.464). This item also showed the weakest validity correlation among the individual items (rho = − 0.46, FDR-adjusted *p* < 0.001). These findings may have two possible explanations. First, they may reflect limitations in the ability of a single doorway-passage trial to consistently elicit FOG. Although walking through a doorway is an established FOG-provoking task, Cowie et al. reported that it failed to elicit FOG in approximately half of the participants with FOG [24]. A recent systematic review found that turning, particularly a 360° turn, was the most effective trigger for eliciting FOG, followed by walking through a doorway and dual tasking [25]. These findings suggest that a single trial of the walking-through-doorway item may have limited sensitivity for eliciting FOG. Second, unlike other items, which are primarily based on clearly timed performance, this item requires raters to judge freezing or festination during doorway passage. FOG is a complex and episodic phenomenon, and previous studies have shown that visual scoring of freezing episodes can involve between-rater variability. Therefore, the relatively lower agreement for this item may reflect the inherent ambiguity of visually identifying freezing episodes [26–28]. Accordingly, interpretation of this item may require caution, particularly when scored by raters with limited experience in assessing FOG.

### Floor and ceiling effects

Consistent with the Brazilian validation study of the LPA [13], we found ceiling effects for the total LPA score and section-level scores, despite the absence of a clear predominance of patients with mild global motor severity based on the MDS-UPDRS Part 3 (range: 3–98, skewness: 0.917). The marked ceiling effect in the gait mobility section may reflect the fact that several items can be completed without apparent difficulty by participants with relatively preserved functional gait, together with the limited ability of the four-point item scoring system to discriminate at the higher end of performance. Given the observed ceiling effects, high LPA scores should be interpreted with caution, particularly in patients with relatively preserved functional mobility, even when global motor severity does not appear to be predominantly mild. Nevertheless, based on previous reviews [6, 29], this limitation may be balanced against the practical advantages of the LPA as a quick and clinically feasible scale for assessing gait and bed mobility in daily practice.

### Limitations

This study has several limitations. First, the modest sample size may limit the generalizability and precision of the psychometric analyses, particularly at the item level. Although the study included a broad range of motor severity (MDS-UPDRS Part III scores, 3–98), no participants with H&Y stage 1 were enrolled, likely reflecting the rehabilitation-based recruitment setting. Second, inter-rater reliability was evaluated in a non-random subsample of 24 participants from a single center. This subsample differed from the remaining participants in levodopa equivalent daily dose and MDS-UPDRS Part II scores, potentially limiting the generalizability of the reliability estimates. Third, the observed ceiling effects, particularly in the gait mobility section, may have reduced discrimination among participants with milder mobility limitations and influenced the observed correlations. Fourth, the participants were recruited from two centers with different clinical profiles. Although sensitivity analyses showed that adjustment for recruitment center resulted in only minimal changes in the construct-validity correlations, residual center-related factors or rater-related measurement variability cannot be completely ruled out. Finally, although the clarification of the maximum-score category for the timed up-and-go item did not alter existing score boundaries, future cross-cultural comparisons should verify how completion times below 10 s were handled in each version. Future studies should address these limitations using larger and more diverse multicenter samples and standardized rater training across sites.

## Conclusion

This study developed a Japanese version of the Lindop Parkinson’s Disease Mobility Assessment (LPA) and examined its validity and reliability in 62 patients with PD. The total LPA score, as well as the gait and bed mobility section scores, showed strong-to-very strong associations with axial motor symptoms and relevant clinical scales, reflecting good construct validity of the Japanese version of the LPA. Reliability analyses demonstrated excellent internal consistency in the full sample (*n* = 62) and excellent inter-rater reliability for the total score in the subsample (*n* = 24), while item-level agreement demonstrated wider variability (quadratic weighted kappa coefficients ranged from 0.46 to 1.00, all *p* ≤ 0.009). These findings suggest that the Japanese version of the LPA is a clinically useful tool for assessing gait and bed mobility difficulties in patients with PD. Future studies are warranted to investigate its responsiveness and applicability among individuals with more advanced functional impairment.

## Supplementary Information

Below is the link to the electronic supplementary material.

Supplementary Material 1

Supplementary Material 2

## Data Availability

The datasets generated and analyzed during the current study are not publicly available because of ethical restrictions but may be made available from the corresponding author on reasonable request, within the limits of participants’ consent.

## Declarations

### Study approval

The study protocol was approved by the Ethics Committee of Osaka University Hospital (No. 22311), the Hyogo Prefectural Rehabilitation Hospital at Nishi-Harima (No. R070513), and Hokkaido Neurological Hospital (No. 2025Nov19-9th).

### Pre-registration

The study was pre-registered in the University Hospital Medical Information Network Clinical Trials Registry (UMIN-CTR; No. UMIN000043418).
