# Supplementary material for: Japanese translation of lindop Parkinson’s disease mobility assessment (LPA): validity and reliability study
Source: Neurol Sci. 2026 Sep 8;47(10):765. doi: 10.1007/s10072-026-09370-0 (PMC13553700; doi:10.1007/s10072-026-09370-0)
Supplement: Supplementary file 1 [file 10072_2026_9370_MOESM1_ESM.pdf]

リンドップ・パーキンソン病アセスメントスケール（ＬＰＡ）

評価日：

氏名：

評価時間：

歩行時の補助器：

ID：

最終服薬の時間：

歩行	**1.** 座位からの立ち上がり	介助は不要で容易<br>介助は不要だが努力性<br>１回の介助<br>２回以上の介助/不可能	3<br>2<br>1<br>0	3<br>2<br>1<br>0
	**2.** 上肢の支えがない立位保持の時間	60 秒以上<br>45～59 秒<br>30～44 秒<br>0～29 秒	3<br>2<br>1<br>0	3<br>2<br>1<br>0
	**3.** タイムドアップ＆ゴーテスト	0～20 秒<br>21～35 秒<br>36～60 秒<br>60 秒超	3<br>2<br>1<br>0	3<br>2<br>1<br>0
	**4.** 右へ**180** 度の方向転換	4～6 歩<br>7～8歩<br>9～10歩<br>11歩以上	3<br>2<br>1<br>0	3<br>2<br>1<br>0
	**5.** 左へ**180** 度の方向転換	4～6 歩<br>7～8歩<br>9～10歩<br>11歩以上	3<br>2<br>1<br>0	3<br>2<br>1<br>0
	**6.** 戸口の通り抜け	すくみなし/加速歩行なし<br>加速歩行あり<br>すくみ足が１回<br>すくみ足が２回以上	3<br>2<br>1<br>0	3<br>2<br>1<br>0
		合計		
ベッド動作	**1.** 端座位から臥位	介助不要で容易(５ 秒以内)<br>介助不要だが努力性(６秒以上)<br>１回の介助<br>２回の介助/不可能	3<br>2<br>1<br>0	3<br>2<br>1<br>0
	**2.** 左側への寝返り	介助不要で容易(５ 秒以内)<br>介助不要だが努力性(６秒以上)<br>１回の介助<br>２回の介助/不可能	3<br>2<br>1<br>0	3<br>2<br>1<br>0
	**3.** 右側への寝返り	介助不要で容易(５ 秒以内)<br>介助不要だが努力性(６秒以上)<br>１回の介助<br>２回の介助/不可能	3<br>2<br>1<br>0	3<br>2<br>1<br>0
	**4.** 臥位からベッド端座位	介助不要で容易(５ 秒以内)<br>介助不要だが努力性(６秒以上)<br>１回の介助<br>２回の介助/不可能	3<br>2<br>1<br>0	3<br>2<br>1<br>0
		合計		

日本版 Lindop Parkinson's Disease Mobility Assessment (LPA)

署名

Copyright©2003Derby Hospitals NHS Foundation Trust.
