# Supplementary material for: Japanese translation of lindop Parkinson’s disease mobility assessment (LPA): validity and reliability study
Source: Neurol Sci. 2026 Sep 8;47(10):765. doi: 10.1007/s10072-026-09370-0 (PMC13553700; doi:10.1007/s10072-026-09370-0)
Supplement: Supplementary file 2 [file 10072_2026_9370_MOESM2_ESM.docx]

**eTable 1. Comparison of demographic and clinical characteristics between the video-based inter-rater reliability subsample and the remaining participants**

|  | **Subsample (n = 24)** | |  | **Remaining participants (n = 38)** | | p-value |
| --- | --- | --- | --- | --- | --- | --- |
|  | Mean | Standard deviation  (min, max) |  | Mean | Standard deviation (min, max) |  |
| Age (years) | 75.8 | 6.9 (60, 88) |  | 74.6 | 7.5 (55, 89) | 0.62 |
| Sex (women/men) | 16/8 | |  | 18/20 | | 0.19 |
| Disease duration (years) | 7.7 | 3.6 (1, 15) |  | 9.2 | 5.9 (0, 25) | 0.45 |
| Hoehn and Yahr stage (2/3/4/5) | n = 6/11/7/0 | |  | n = 8/19/7/4 | | 0.36 |
| LPA total score (range 0–30) | 23.9 | 5.8 (10, 30) |  | 22.1 | 10.1 (0, 30) | 0.74 |
| Gait mobility section score (range 0–18) | 14.5 | 4.9 (2, 18) |  | 12.9 | 6.6 (0, 18) | 0.50 |
| Bed mobility section score (range 0–12) | 9.4 | 1.5 (7, 12) |  | 9.2 | 3.8 (0, 12) | 0.18 |
| Levodopa equivalent daily dose (mg/day) | 544.1 | 253.5 (100, 1120) |  | 737.2 | 342.3 (100, 1450) | 0.03 |
| MDS-UPDRS Part 2 total (range 0–52) | 12.1 | 8 (1, 30) |  | 19.7 | 13.5 (0, 52) | 0.03 |
| Item 2.9, Turning in bed (range 0–4) | 1.1 | 0.9 (0, 4) |  | 1.4 | 1.4 (0, 4) | 0.81 |
| Item 2.11, Getting out of bed/chair (range 0–4) | 1.1 | 1 (0, 4) |  | 1.7 | 1.3 (0, 4) | 0.13 |
| MDS-UPDRS Part 3 total (range 0–132) | 31 | 12 (3, 53) |  | 38.5 | 19.9 (8, 98) | 0.23 |
| Item 3.9, Arising from chair (range 0–4) | 0.7 | 0.7 (0, 2) |  | 1.2 | 1.6 (0, 4) | 0.95 |
| Item 3.10, Gait (range 0–4) | 1.6 | 1 (0, 3) |  | 1.6 | 1.5 (0, 4) | 0.61 |
| Item 3.11, Freezing of gait (range 0–4) | 0.5 | 0.8 (0, 3) |  | 0.9 | 1.4 (0, 4) | 0.47 |
| Item 3.12, Postural Stability (range 0–4) | 2.1 | 1.3 (0, 4) |  | 2.1 | 1.5 (0, 4) | 0.96 |
| Axial subscore (range 0–24) | 7.1 | 3.6 (0, 14) |  | 8.8 | 6.8 (0, 24) | 0.82 |
| Bradykinesia subscore (range 0–48) | 13.8 | 7.4 (2, 28) |  | 16.7 | 10.5 (0, 47) | 0.36 |
| Rigidity subscore (range 0–20) | 7.3 | 2.7 (0, 11) |  | 9.0 | 3.6 (3, 15) | 0.09 |
| Berg Balance Scale (range 0–56) | 47.2 | 8.5 (26, 56) |  | 38.8 | 20.6 (0, 56) | 0.64 |
| Mini-Mental State Examination (range 0–30) | 27.0 | 1.9 (23, 30) |  | 25.6 | 4.6 (11, 30) | 0.82 |

Values are presented as mean ± standard deviation (minimum, maximum), except for sex and Hoehn and Yahr stage, which are presented as counts. P-values were calculated using the Wilcoxon rank-sum test for continuous variables and Fisher’s exact test for categorical variables.

**eTable 2.** **Center-specific demographic and clinical characteristics**

|  | **Hyogo center (n = 30)** | |  | **Hokkaido center (n = 32)** | | Standardized mean differences |
| --- | --- | --- | --- | --- | --- | --- |
|  | Mean | Standard deviation  (min, max) |  | Mean | Standard deviation  (min, max) |  |
| Age (years) | 74.0 | 7.2 (55, 85) |  | 76.1 | 7.2 (60, 89) | 0.291 |
| Sex (women/men) | 14/16 | |  | 20/12 | | 0.322 |
| Disease duration (years) | 8.1 | 5.6 (0, 23) |  | 9.1 | 4.7 (1, 25) | 0.186 |
| Hoehn and Yahr stage (2/3/4/5) | n = 8/19/3/0 | |  | n = 6/11/11/4 | | 0.935 |
| LPA total score (range 0–30) | 26.6 | 4.3 (13, 30) |  | 19.2 | 10.2 (0, 30) | 0.945 |
| Gait mobility section score (range 0–18) | 15.8 | 3.5 (4, 18) |  | 11.5 | 7.1 (0, 18) | 0.764 |
| Bed mobility section score (range 0–12) | 10.8 | 1.6 (6, 12) |  | 7.8 | 3.4 (0, 12) | 1.161 |
| Levodopa equivalent daily dose (mg/day) | 719.0 | 353.2 (100, 1450) |  | 613.8 | 288.9 (100, 1210) | 0.326 |
| MDS-UPDRS Part 2 total (range 0–52) | 14.5 | 8.1 (0.0, 29) |  | 18.9 | 14.8 (1, 52) | 0.372 |
| Item 2.9, Turning in bed (range 0–4) | 0.8 | 0.8 (0, 3) |  | 1.6 | 1.4 (0, 4) | 0.705 |
| Item 2.11, Getting out of bed/chair (range 0–4) | 1.1 | 0.9 (0, 3) |  | 1.8 | 1.5 (0, 4) | 0.515 |
| MDS-UPDRS Part 3 total (range 0–132) | 31.0 | 13.2 (8, 60) |  | 39.9 | 20.0 (3, 98) | 0.525 |
| Item 3.9, Arising from chair (range 0–4) | 0.5 | 0.9 (0, 3) |  | 1.5 | 1.5 (0, 4) | 0.809 |
| Item 3.10, Gait (range 0–4) | 1.0 | 1.1 (0, 4) |  | 2.2 | 1.3 (0, 4) | 0.923 |
| Item 3.11, Freezing of gait (range 0–4) | 0.4 | 0.7 (0, 3) |  | 1.1 | 1.4 (0, 4) | 0.617 |
| Item 3.12, Postural Stability (range 0–4) | 1.5 | 1.3 (0, 4) |  | 2.6 | 1.4 (0, 4) | 0.763 |
| Axial subscore (range 0–24) | 6.1 | 4.4 (0, 18) |  | 10.1 | 6.3 (0, 24) | 0.736 |
| Bradykinesia subscore (range 0–48) | 13.0 | 7.2 (0, 29) |  | 18.0 | 10.7 (2, 47) | 0.553 |
| Rigidity subscore (range 0–20) | 8.3 | 3.6 (3, 15) |  | 8.4 | 3.1 (0, 15) | 0.012 |
| Berg Balance Scale (range 0–56) | 48.4 | 9.2 (15, 56) |  | 39.4 | 18.0 (0, 56) | 0.633 |
| Mini-Mental State Examination (range 0–30) | 26.9 | 2.4 (20, 30) |  | 25.4 | 4.7 (11, 30) | 0.409 |

Values are presented as mean ± standard deviation, except for sex and Hoehn and Yahr stage, which are presented as counts. Standardized mean differences were calculated for continuous and binary variables, and a generalized standardized difference was calculated for Hoehn and Yahr stage. Note: These standardized differences are presented for descriptive purposes only to transparently characterize the center-specific samples, as described in the Methods of the main text. The potential influence of recruitment center on construct validity was examined separately using center-adjusted partial Spearman correlations (**eTable 3**).

Abbreviations: FDR = false discovery rate; LPA = Lindop Parkinson’s Disease Mobility Assessment; MDS-UPDRS = Movement Disorder Society-sponsored revision of the Unified Parkinson’s Disease Rating Scale.

**eTable 3. Sensitivity analysis of construct validity using partial Spearman correlations adjusted for recruitment center (n = 62)**

| **LPA score** | **Validation measure** | **Unadjusted Spearman’s rho** | **Center-adjusted partial Spearman's rho** | **FDR-adjusted**  **p value** |
| --- | --- | --- | --- | --- |
| **LPA total score** | MDS-UPDRS Part 3 total score | −0.74 | −0.73 | < 0.0001 |
|  | Axial subscore | −0.83 | −0.80 | < 0.0001 |
|  | Bradykinesia subscore | −0.70 | −0.69 | < 0.0001 |
|  | BBS | 0.75 | 0.70 | < 0.0001 |
| **Gait section score** | Axial subscore | −0.79 | −0.76 | < 0.0001 |
|  | MDS-UPDRS item 3.10, gait | −0.70 | −0.66 | < 0.0001 |
|  | MDS-UPDRS item 3.11, freezing of gait | −0.52 | −0.48 | < 0.001 |
|  | BBS | 0.70 | 0.65 | < 0.0001 |
| Item 1: Sit-to-stand | MDS-UPDRS item 3.9, arising from chair | −0.80 | −0.79 | < 0.0001 |
| Item 2: Unsupported standing | MDS-UPDRS item 3.12, postural stability | −0.57 | −0.50 | < 0.0001 |
| Item 3: Timed Up and Go | BBS | 0.75 | 0.70 | < 0.0001 |
| Item 4: 180-degree turn, right | MDS-UPDRS item 3.10, gait | −0.60 | −0.56 | < 0.0001 |
| Item 4: 180-degree turn, right | MDS-UPDRS item 3.11, freezing of gait | −0.50 | −0.47 | < 0.001 |
| Item 5: 180-degree turn, left | MDS-UPDRS item 3.10, gait | −0.60 | −0.56 | < 0.0001 |
| Item 5: 180-degree turn, left | MDS-UPDRS item 3.11, freezing of gait | −0.47 | −0.43 | < 0.001 |
| Item 6: Walking through doorway | MDS-UPDRS item 3.11, freezing of gait | −0.46 | −0.41 | < 0.001 |
| **Bed mobility section score** | Axial subscore | −0.71 | −0.67 | < 0.0001 |
|  | Bradykinesia subscore | −0.63 | −0.62 | < 0.0001 |
|  | Rigidity subscore | −0.35 | −0.40 | < 0.01 |
| Item 1: Sit-to-lie | MDS-UPDRS item 2.11, getting out of bed/chair | −0.49 | −0.47 | < 0.001 |
| Items 2 and 3: Turning in bed | Axial subscore | −0.63 | −0.56 | < 0.0001 |
| Items 2 and 3: Turning in bed | MDS-UPDRS item 2.9, turning in bed | −0.60 | −0.55 | < 0.0001 |
| Item 4: Lie-to-sit | MDS-UPDRS item 2.11, getting out of bed/chair | −0.47 | −0.44 | < 0.001 |

Unadjusted Spearman correlation coefficients correspond to those reported in Table 2. Center-adjusted partial Spearman correlation coefficients were calculated controlling for recruitment center. P values for the center-adjusted correlations were corrected for multiple comparisons using the false discovery rate method.

Abbreviations: MDS-UPDRS = Movement Disorder Society-sponsored revision of the Unified Parkinson’s Disease Rating Scale, BBS = Berg Balance Scale, FOG = freezing of gait, Getting out of bed/chair = Getting out of bed, a car or a deep chair, FDR = the false discovery rate.

**Gait mobility section**

**Bed mobility section**

**eFig. 1 Correlations between section-level LPA scores and relevant clinical measures**

The x-axis shows the section level LPA score, and the y-axis represents each relevant clinical measure. Each point represents an individual participant’s raw data, and points were slightly jittered horizontally to improve the visibility of overlapping observations. The blue line represents a fitted linear trend line for visualization, and gray shaded areas indicate 95% confidence intervals around the fitted line. Spearman’s rho and p-values are summarized in Table 2.
